# A taxon-centered review of bacterial shifts in psychiatric disorders

**DOI:** 10.3389/fpsyt.2026.1702172

**Published:** 2026-03-02

**Authors:** Răzvan-Ioan Papacocea, Adela-Magdalena Ciobanu

**Affiliations:** 1 Department of Psychiatry, Faculty of Medicine, Carol Davila University of Medicine and Pharmacy, Bucharest, Romania; 2 Department of Psychiatry, Prof. Dr. Alexandru Obregia Clinical Hospital of Psychiatry, Bucharest, Romania

**Keywords:** anxiety, autism spectrum disorder, depression, gut microbiota, gut-brain axis, psychiatric disorder, schizophrenia

## Abstract

**Background:**

Psychiatric conditions rank among the leading causes of disability worldwide, with their burden steadily increasing in recent years. Recent research highlights the gut-brain axis as a pivotal pathway in mental health, implicating gut microbiota shifts in conditions such as depression, anxiety, schizophrenia, bipolar disorder, autism spectrum disorder, Alzheimer’s disease and others. However, most reviews remain diagnosis-centered.

**Methods:**

We conducted a structured literature review of articles published between January 2015 and July 2025 in PubMed, Scopus, and Web of Science. We included both human and animal studies that reported taxonomic changes in gut microbiota associated with psychiatric or neurodevelopmental disorders. Editorials, conference abstracts, and studies lacking full-text availability, not addressing psychiatric outcomes or specific taxonomic data were excluded. Thus, data on bacterial taxa reported as increased or decreased versus controls were extracted and reorganized into a taxon-centered database.

**Results:**

The analysis suggested distinct yet overlapping microbial alterations across psychiatric conditions. Taxa such as *Coprococcus* and *Faecalibacterium* were repeatedly reported as decreased in multiple disorders, suggesting a possible reduction in taxa commonly associated with anti-inflammatory functions, while *Bacteroides*, *Lactobacillus*, and *Clostridium* were reported in context-dependent associations. Some genera (e.g., *Desulfovibrio*, *Klebsiella*, *Methanobrevibacter*) were reported as enriched across disorders, potentially reflecting shared inflammatory-related profiles. This transdiagnostic mapping highlights microbial taxa that recur across psychiatric conditions and may represent candidates for further investigation.

**Conclusion:**

By changing the perspective from diagnosis to taxon-centered analysis, this review suggests microbial signatures that appear across psychiatric diseases, supporting the possibility of shared pathophysiological pathways. Given the largely associative nature of the available data, these findings should be interpreted cautiously and may help guide future research exploring the role of the gut microbiota in mental health.

## 1 Introduction

Mental health disorders are among the leading causes of disability worldwide and their prevalence has risen steadily in recent decades. According to the World Health Organization, nearly one in eight adults globally live with a mental disorder, a number that surged further after the COVID-19 pandemic. These conditions are often underdiagnosed and stigmatized, particularly in low- and middle-income countries, amplifying their global burden (1, 2).

The link between gut and brain health has been suspected for centuries. Hippocrates’ dictum that “all diseases begin in the gut” and Mechnikov’s observations on fermented foods and longevity anticipated this connection (3, 4). While these early views lacked rigorous evidence, modern studies now implicate intestinal dysbiosis in depression, anxiety, schizophrenia, and bipolar disorder (5, 6). Mechanistic findings highlight inflammation, neurotransmission, and stress reactivity as key mediators of this relationship (7, 8). Because psychiatric illnesses are multifactorial (9), diagnosis-centered reviews may overlook microbial patterns that cut across disorders. A taxon-centered approach offers a complementary perspective by examining bacterial taxa directly rather than focusing on individual diagnoses. Accordingly, this review maps reported bacterial alterations across psychiatric disorders and aims to identify microbial signatures that may be relevant to disease-related processes. This perspective may enhance understanding of shared biological pathways and help inform future research on microbiota-related mechanisms in psychiatry.

## 2 Materials and methods

This review was developed based on a structured analysis of published literature on the microbiota-gut-brain axis in psychiatric disorders.

### 2.1 Eligibility criteria

Studies were eligible if they: (i) were published between January 2015 and July 2025, (ii) reported quantitative alterations in gut microbiota composition in association with psychiatric or neurodevelopmental disorders (depression, anxiety disorders, schizophrenia, bipolar disorder, post-traumatic stress disorder, autism spectrum disorder, Alzheimer’s disease, obsessive-compulsive disorder, attention deficit hyperactivity disorder, and eating disorders), (iii) included either human subjects or preclinical animal models, (iv) represented original research (clinical or preclinical) or reviews, and (v) were available in English as full-text publications.

Exclusion criteria were: (i) studies published before 2015 (ii) commentaries, editorials, conference abstracts, or case reports, (iii) studies that did not report microbiota changes (no specific bacterial phylum, genus, or species reported), and (iv) studies limited to non-psychiatric conditions.

### 2.2 Information sources

The literature search was conducted in PubMed, Scopus, and Web of Science, with the last update performed in July 2025. In addition, epidemiological data were retrieved from the World Health Organization (WHO) and the Institute for Health Metrics and Evaluation (IHME).

### 2.3 Search strategy

Search terms combined microbiota-related keywords with psychiatric disorders, using Boolean operators. Filters were applied to restrict the search to articles published in English and within the time frame 2015-2025. “An illustrative query was (“gut microbiota” OR “gut microbiome”) AND (“ depression” OR” anxiety” OR …).

### 2.4 Selection process

**Figure 1:**
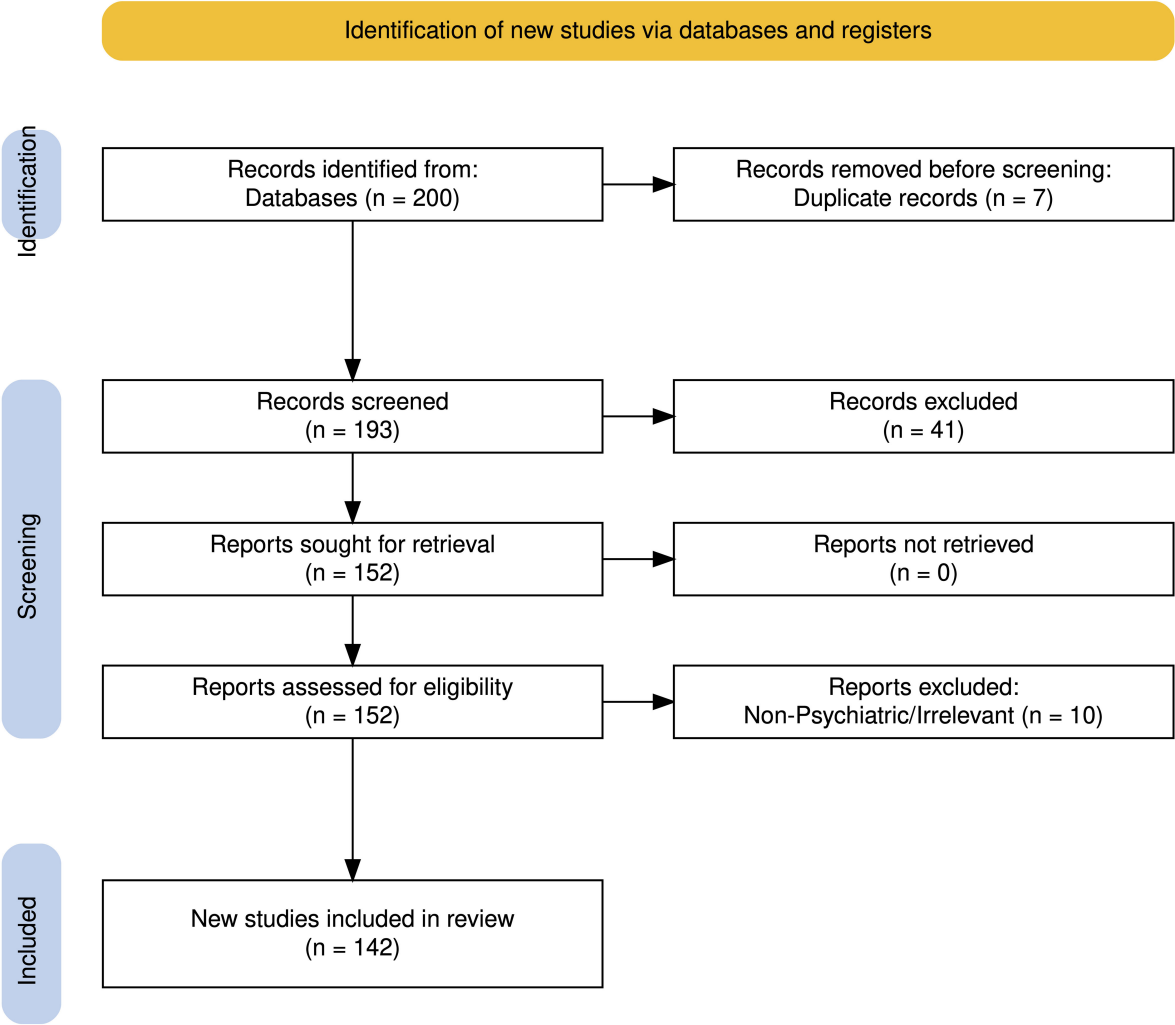
A total of 200 records were identified from PubMed, Scopus, and Web of Science. After removal of 7 duplicates, 193 records were screened based on title and abstract. Of these, 41 were excluded as irrelevant. Full-text assessment was performed for 152 reports, of which 10 were excluded (due to irrelevance OR non-psychiatric focus),. Finally, 142 studies met the eligibility criteria and were included in the review.

Titles and abstracts were screened to identify potentially relevant studies. Full texts of eligible records were retrieved and reviewed for final inclusion. Duplicates were removed manually. The overall process followed PRISMA guidelines, and a flow diagram (**Figure 1**) summarizes the number of studies identified, screened, excluded, and included.

### 2.5 Data collection process

For each eligible study, data were independently extracted into a structured table (**Supplementary Tables 1A, B**). Extracted variables included: psychiatric disorder studied, type of model (human or animal), bacterial taxa increased or decreased in patients compared to healthy controls, and bibliographic reference.

### 2.6 Data items

The main outcome of interest was the direction of change in bacterial taxa (increase or decrease) in psychiatric disorders. Additional extracted information included: disorder type, human vs. animal model, and publication details.

### 2.7 Study risk of bias assessment

Although no formal risk-of-bias tool was applied, study quality was indirectly addressed through strict inclusion and exclusion criteria, ensuring that only original research or reviews articles available in English as full-text publications were included.

### 2.8 Synthesis methods

**Figure 2:**
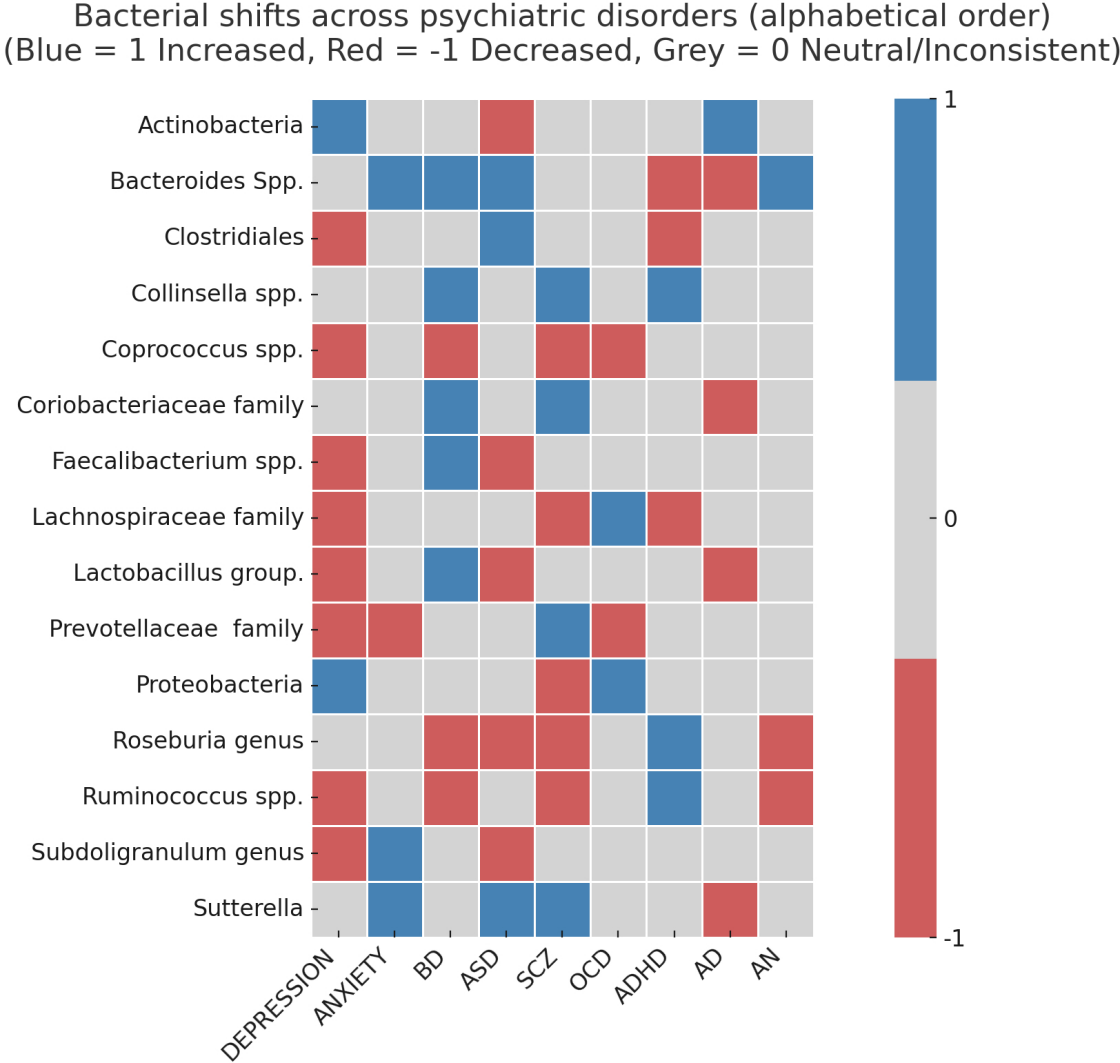
Summary of bacterial shifts across major psychiatric disorders. The heatmap illustrates directionality of microbial changes (blue = increased abundance, red = decreased abundance, grey = inconsistent or neutral findings) across psychiatric conditions. Several taxa, including Bacteroides, Proteobacteria, and Lactobacillus, show disorder-specific alterations, while others such as Coprococcus display consistent reductions across multiple diagnoses, suggesting loss of anti-inflammatory bacteria may represent a transdiagnostic feature of psychiatric pathology. BD, Bipolar Disease; ASD, Autism Spectrum Disorder; SCZ, Schizophrenia; OCD, Obsessive-Compulssive Disorder; ADHD, Attention deficit hyperactivity disorder; AD Alzheimer Disease; AN, Anorexia Nervosa.

Given the descriptive and taxonomy-centered nature of this review, no formal effect size estimation, risk-of-bias assessment, or certainty grading was performed. Instead, data were reorganized by bacterial taxonomy rather than by disorder, allowing the identification of transdiagnostic microbial patterns. Results were summarized in **Supplementary Tables 1A, B** and further visualized through a summarized heatmap (**Figure 2**).

## 3 Review content

### 3.1 The gut-brain axis

**Figure 3:**
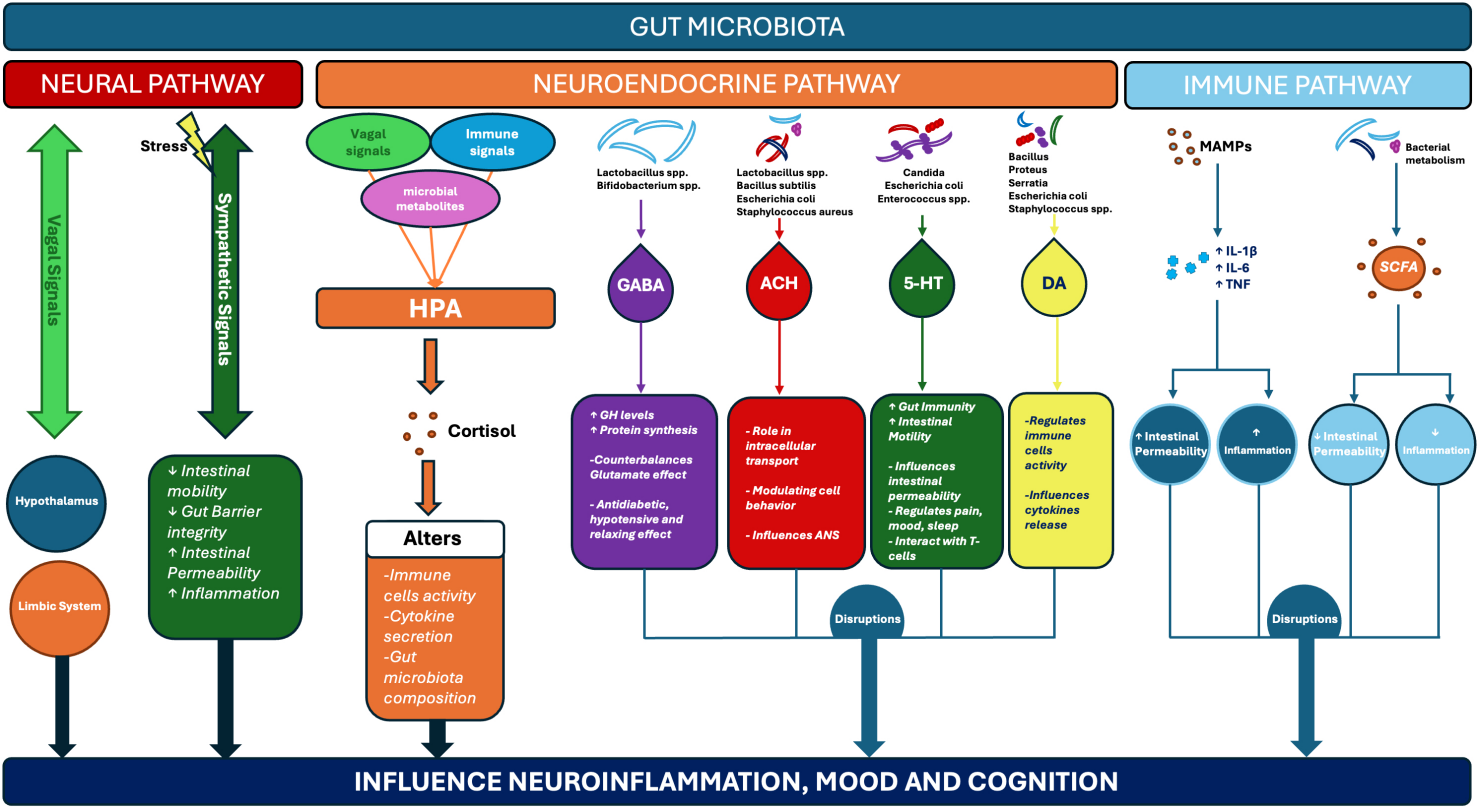
Mechanistic pathways linking gut microbiota to neuropsychiatric outcomes. Gut microbiota influence the brain through three interconnected routes. The neural pathway involves vagal signaling, which projects to the hypothalamus and limbic system and thereby transmits microbial and inflammatory information to emotion-related brain regions, as well as sympathetic signaling, which modulates gut motility, barrier integrity, permeability, and inflammatory responses. The neuroendocrine pathway is mediated by the hypothalamic-pituitary-adrenal (HPA) axis, which regulates cortisol release and stress responses, together with microbially derived neurotransmitters. Among these, gamma-aminobutyric acid (GABA) counterbalances the excitatory effects of glutamate, stimulates protein synthesis, and exerts relaxing, antidiabetic, and hypotensive effects; acetylcholine (ACH) modulates intracellular transport, cell behavior, and autonomic nervous system activity; serotonin (5-HT) regulates gut immunity, intestinal permeability, pain, sleep, and mood, and also interacts with T cells; while dopamine (DA) regulates immune cell stimulation and cytokine release, and in the brain modulates nitric oxide synthesis and microglial migration. The immune pathway is activated by microbial-associated molecular patterns (MAMPs), which induce pro-inflammatory cytokines such as interleukins (IL-1β, IL-6) and tumor necrosis factor (TNF), leading to increased intestinal permeability and inflammation. In contrast, bacterial metabolites such as short-chain fatty acids (SCFAs), strengthen barrier integrity and reduce inflammation. Upward arrows in the figure indicate stimulation or increase, whereas downward arrows indicate inhibition or reduction. Together, these interconnected mechanisms converge on neuroinflammation, mood, cognition, and psychiatric symptoms.

The gut-brain axis (GBA) is a bidirectional communication network linking the central nervous system (CNS) and the intestinal microbiota (10, 11). Physiologically, it includes four major components: the Enteric Nervous System (12), the Intestinal Microbiota (estimated to weigh 1–2 kg) (13), the Vagus Nerve, and the Brain (14). Increasing evidence suggests an involvement in both brain development and psychiatric disorders (15, 16). Communication occurs through neural, endocrine, immune, and metabolic pathways (17, 18) potentially influencing neurodevelopment, neurotransmitter function, and behavior (19) (**Figure 3**). Imbalances in the gut microbiota (commonly referred to dysbiosis) have been linked to several psychiatric conditions (20), including major depressive disorder (21–24) schizophrenia (25, 26), bipolar disorder (27, 28), autism spectrum disorder (ASD) (29, 30) anxiety (31, 32) ADHD (10, 33) and Alzheimer’s disease (34). Associations have also been reported in neurological disorders such as Parkinson’s disease (35) and Tourette syndrome (36). Key mediators include microbial metabolites such as short-chain fatty acids (37), neurotransmitters, immune pathways, and hypothalamic-pituitary-adrenal (HPA) axis signaling (38, 39). Animal and early human studies suggest that interventions targeting the gut microbiota (for example probiotics (40, 41), prebiotics, or fecal microbiota transplantation) may be associated with changes in psychiatric or behavioral outcomes (42, 43). However, clinical translation remains limited by challenges in establishing causality, elucidating mechanisms, and bridging findings from models to patients (44, 45). Emerging approaches, such as probiotics, prebiotics, synbiotics, postbiotics, and fecal microbiota transplantation (FMT), are currently being investigated in psychiatric research, with their clinical relevance and mechanisms of action still under evaluation (46, 47).

### 3.2 Neural pathways: ENS, ANS, vagal and spinal afferent signaling

The enteric nervous system (ENS) and autonomic nervous system (ANS)- parasympathetic (vagal) and sympathetic pathways- form key routes of gut-brain communication (48). Information through ENS and ANS flows bidirectionally (49). Stress-induced sympathetic activity reduces gut motility and barrier integrity (50, 51), disrupts colonization of the commensal bacteria (52), and increases intestinal permeability (53). Together, these changes have been associated with processes, relevant to stress-related and psychiatric phenotypes. Vagal fibers transmit microbial and inflammatory signals from the gut to emotion-related brain regions, including the hypothalamus (39, 54) and the limbic system (55). In experimental models, Bifidobacterium longum and other bacterial species were associated with differences in these circuits and stress-related behaviors (56–58). Spinal afferents also contribute, as cytokines released in the gut mucosa can activate pain and emotion-processing pathways (59–61). (**Figure 3**).

### 3.3 HPA axis and neuroendocrine modulation

Gut microbiota interact with the HPA axis through three main pathways: vagal signaling (62), immune signaling (63), and microbial metabolites (64). The activity of the HPA axis, in turn, affects gut motility and microbial diversity (65–67), thereby shaping neuroendocrine regulation. Dysregulation of this axis has been associated with anxiety (68) and depression (69). Cortisol plays a central role in these mechanisms. It regulates immune cell activity (70), modulates cytokine secretion (70, 71), and indirectly alters the composition and function of the intestinal microbiota (54, 72–74). In addition, several gut bacteria species synthesize neuroactive compounds (75) associated with communication between the intestine and the CNS (**Figure 3**).

#### 3.3.1 Gamma-aminobutyric acid

is an inhibitory neurotransmitter that can be produced by *Lactobacillus* and *Bifidobacterium* spp. In mammals, it counterbalances the excitatory effects of glutamate. Within the central nervous system, GABA increases growth hormone levels and stimulates protein synthesis (76–78). It also exerts systemic effects, including diuretic and hypotensive actions, lowering blood pressure (79). Additionally, GABA has been linked to antidiabetic properties (80, 81) and relaxing effects (77, 82) (**Figure 3**).

#### 3.3.2 Acetylcholine

is synthesized from choline and can be released by several bacteria, including Lactobacillus spp., Bacillus subtilis, Escherichia coli, Staphylococcus aureus (83). It plays a role in intracellular transport (84) and in modulating cell behavior (85). Once released, acetylcholine influences the autonomic nervous system (86) (**Figure 3**).

#### 3.3.3 Serotonin

can be produced by Escherichia, Candida, and Enterococcus spp (87). It contributes to the gut immune development (88), immune tolerance (89), gut barrier permeability (90, 91) and intestinal motility (92). Around 95% of the body’s serotonin is synthesized in the gut (93), mainly by enterochromaffin cells (ECL cells), microbiota, and enteric nervous system neurons. Serotonin regulates pain, mood and sleep. Alterations in gut microbiota composition have been associated with depression, anxiety, and other neuropsychiatric conditions (94). Central serotonin is essential for mood and behavior regulation and helps maintain energy homeostasis by suppressing appetite. In experimental models, serotonin can directly interact with T lymphocytes (T cells), increase intracellular levels of indole-3-acetaldehyde, suppress the mechanistic target of rapamycin (mTOR) activation and stimulates the differentiation of regulatory T cells in a murine model (92–96) (**Figure 3**).

#### 3.3.4 Dopamine & others

In the gut microbiota, dopamine can be produced by species such as Staphylococcus, Bacillus, Proteus, Serratia, and Escherichia. Dopamine regulates immune cell activity and cytokine release by T cells. In the brain, it modulates nitric oxide synthesis and migration of microglia (97, 98) (**Figure 3**). Norepinephrine can be secreted by Bacillus and Saccharomyces spp (99). Chronic stress impairs the integrity of the blood-brain barrier (100). Microbially derived substances exert both local and systemic effects, influencing the gut directly while also modulating brain activity at distance (101, 102). Stress-induced inflammation has been associated with disruptions in brain function and with anxiety/depression-related phenotypes. Key mechanistic pathways include short-chain fatty acid (SCFA) production, tryptophan metabolism, vagal nerve signaling, and microbial pattern recognition. Evidence from germ-free animal models, as well as interventions such as probiotics, prebiotics, and fecal microbiota transplants (FMT), supports the potential relevance of these pathways for further mechanistic investigation (103). Moreover, several studies report that excessive glutamate entry into the central nervous system disrupts the glutamate balance. Excessive glutamate accumulation in the central nervous system has been associated with inflammation and neurotoxic effects in experimental settings (104–106).

### 3.4 Neuroimmune interactions

Microbial Associated Molecular Patterns (MAMPs) are conserved molecular structures present in bacteria, fungi and viruses, but not in human cells (107). They signal microbial presence and include lipopolysaccharides (LPS), peptidoglycans, flagellin and others. These molecules trigger the release of proinflammatory cytokines such as interleukin-1β (IL-1β), interleukin-6 (IL-6) and tumor necrosis factor (TNF), which increase the blood-brain barrier permeability and stimulate neuroinflammation (108–110). Microbial metabolites are small molecules produced during intestinal digestion and bacterial metabolism, that have been implicated in interactions with brain metabolism and function. Some of them may exert neuroprotective effects (111). SCFA including acetate (112), butyrate (25), and propionate (113) have been involved in blood-brain barrier integrity (114), inflammatory signaling, and neuroendocrine responses (115). Monoamine neurotransmitters such as serotonin (116), dopamine (117), norepinephrine (118), GABA (119), along with tryptophan-kynurenine metabolites (120), generate compounds including indole-3-acetic acid (121), indole-3-propionic acid (122) and tryptamine (123). These compounds have been connected with effects on inflammation, mood and cognition (**Figure 3**).

## 4 Results

By adopting a bacteria-centered approach, this review maps a broad landscape of reported microbial alterations across psychiatric disorders (see **Supplementary Tables 1A, B**; **Figure 2**). Rather than organizing findings by diagnosis, bacterial taxa were grouped based on the direction of reported change (increase or decrease) in patients compared with healthy controls. In total, several hundred taxonomic alterations were extracted from the literature and systematically classified according to taxon, condition, and direction of change, with methodological and cohort details summarized in **Supplementary Tables 1A, B**.

The taxonomic patterns reported in this review were derived from a highly heterogeneous body of literature, including both human observational studies and animal models, with substantial variability in cohort characteristics, medication status, and microbiota profiling methodologies (e.g., 16S rRNA sequencing versus shotgun metagenomics). Human studies included diverse populations, ranging from the first-episode to chronic patients, with medication status variably reported or controlled, while animal studies employed different species and experimental models. Accordingly, the present synthesis is descriptive in nature and reflects reported associations rather than causal or functional relationships. The taxon-centered approach adopted here aims to map convergent patterns across disorders while explicitly acknowledging the methodological and clinical heterogeneity underlying the available data.

This reversed, taxon-centered structure enabled the identification of transdiagnostic patterns across psychiatric disorders. Multiple genera and families were reported as altered across more than one diagnosis, including *Alistipes*, *Bacteroides*, *Clostridiales*, and *Roseburia* (see **Supplementary Tables 1A, B**). For example, *Alistipes* spp. were reported as increased in depression but decreased in Alzheimer’s disease. *Bacteroides* spp. were reported as increased in anxiety, bipolar disorder, and autism spectrum disorder (ASD), while decreased in attention-deficit/hyperactivity disorder (ADHD) and Alzheimer’s disease. Alterations in the *Clostridiales* order included increased levels in ASD and decreased levels in depression and ADHD. *Roseburia* genus levels were reported as increased in ADHD but decreased in bipolar disorder, ASD, schizophrenia, and anorexia nervosa.

Across conditions, several taxa were reported in at least two psychiatric disorders, including *Actinomycetota*, *Bacteroides*, *Blautia*, *Collinsella*, *Coprococcus*, *Firmicutes*, *Lachnospiraceae*, *Lactobacillus*, *Prevotellaceae*, *Ruminococcus*, and *Sutterella* (see **Supplementary Tables 1A, B**). Disorder-specific patterns were also observed. As summarized in **Supplementary Tables 1A, B**, studies in ASD reported increased levels of *Clostridium*, *Desulfovibrio*, and *Sutterella*, alongside decreased levels of *Bifidobacterium*, *Subdoligranulum*, and *Oscillospira*. In schizophrenia, reported increases included *Collinsella*, *Klebsiella*, and *Methanobrevibacter*, whereas decreases were reported for *Blautia*, *Escherichia coli*, and *Roseburia*. Notably, for some taxa, similar directions of change were reported across both human and animal studies, as summarized in **Supplementary Tables 1A, B** [as mentioned in the study of Samulėnaitė et al. (156)].

## 5 Discussion

The results of this review support the idea that psychiatric disorders may involve biological processes extending beyond the central nervous system and include interactions with the gut ecosystem. By focusing on microbial taxa rather than diagnostic categories, this taxon-centered synthesis highlights transdiagnostic microbial signatures that may relate to shared pathophysiological processes across psychiatric conditions. Importantly, the gut-brain axis is not unique to humans; animal studies have also shown associations between gut microbiota composition and behavioral or cognitive phenotypes (124), suggesting that this communication pathway is evolutionarily conserved.

Several taxa identified in this review are known to possess active metabolic capacities. Genera such as *Alistipes*, *Bacteroides*, *Faecalibacterium*, *Lactobacillus*, and *Clostridium* are involved in the production of SCFA and in pathways related to inflammation and neuroactive molecules, including GABA, serotonin, and tryptamine. Alterations in these taxa may therefore be relevant to neuroinflammatory processes, stress-related responses (125), systemic homeostasis (125–127), and gut barrier integrity (128). Notably, some taxa appear to display context-dependent associations. For example, *Bacteroides fragilis* was associated with beneficial effects in ASD animal models when administered as a probiotic, whereas in another study, reduced levels were reported in humans with Alzheimer’s disease (20). Similarly, *Lactobacillus* spp. were reported as decreased in ASD and Alzheimer’s disease but associated with improved depressive symptoms when included in probiotic formulations (20, 126). These observations suggest that host-related factors, such as genetics, age, immune status, diet, and disease context, may shape microbiota-host interactions.

Across studies, findings for individual taxa were not always consistent. Such inconsistencies likely reflect substantial methodological heterogeneity, including differences in sequencing approaches (16S rRNA versus shotgun metagenomics), sequencing depth, taxonomic resolution, and analytical pipelines. Geographic and dietary factors may further contribute to variability, as Western cohorts consuming high-fat, low-fiber diets often display distinct microbial profiles compared with populations consuming fiber-rich diets. These background differences may partly account for divergent findings, particularly for frequently reported taxa.

Despite this heterogeneity, recurring patterns were observed. Reduced abundance of taxa such as *Coprococcus*, commonly described as anti-inflammatory or butyrate-producing taxa, was repeatedly reported across several psychiatric conditions (20, 33). Conversely, genera often associated with pro-inflammatory profiles, including *Desulfovibrio*, *Klebsiella*, and *Methanobrevibacter* (37, 126), were reported as increased. Together, these patterns may reflect a shift toward reduced microbial resilience and altered immune-metabolic balance. At the same time, several taxa, including *Bifidobacterium longum*, *Lactobacillus helveticus* (10), and *Lactobacillus plantarum* (19, 56), have shown beneficial associations with emotional or cognitive outcomes in both human and animal studies, highlighting their potential relevance for future mechanistic and interventional research rather than established therapeutic use.

Clinical studies further underscore the complexity of gut-brain associations. In first-episode psychosis (FEP), increased *Lactobacillus* abundance was reported, with individuals showing the largest deviations exhibiting poorer treatment response at 12 months (129). Bipolar disorder (BD) and schizophrenia spectrum disorders (SSD) have been associated with shifts in taxa such as *Lachnoclostridium* and *Eggerthella* (130). In a cohort of young adults with major depressive disorder (MDD), reductions in *Firmicutes* and particularly *Clostridia* and *Faecalibacterium*, were observed alongside increases in *Bacteroidetes* and *Flavonifractor* (49, 131). Functional predictions suggested reduced short-chain fatty acid pathways, and reported associations with depression severity were independent of medication use (131).

Disorder-specific microbial profiles have also been described. Depression has frequently been associated with reduced microbial diversity, anxiety disorders with fewer SCFA-producing taxa, schizophrenia with endotoxemia-related signatures, and BD with altered Firmicutes/Bacteroides ratios (132, 133). However, divergent findings are common, even within the same diagnostic category. For example, Sanada et al. reported reduced Prevotellaceae, Coprococcus, and Faecalibacterium in MDD (134), whereas other studies observed increased Streptococcus, and Parabacteroides alongside reductions in Faecalibacterium and Bifidobacterium (135). Such discrepancies likely reflect patient-related factors, including medication use, disease stage, and comorbidities.

Network-based analyses also suggest that microbial community structure, rather than individual taxa alone, may be relevant to psychiatric phenotypes. In MDD, co-occurrence network analyses identified *Ruminococcaceae* and *Clostridiales* taxa associated with depression, anxiety, and anhedonia (136). Large population-based studies reported connections between depressive symptom severity and multiple taxa, including *Eggerthella*, *Coprococcus*, *Subdoligranulum*, and *Ruminococcaceae* genera (137). In schizophrenia, increased abundance of *Collinsella, Lactobacillus, Succinivibrio, and Corynebacterium*, alongside reduced *Faecalibacterium and Anaerostipes*, has been reported, with some taxa associated with symptom dimensions such as PANSS scores or negative symptoms (138–140). Altered microbial network organization and reduced competitive interactions among key taxa may contribute to ecological instability and dysbiosis (141).

Beyond the gut, differences in oropharyngeal microbiota have also been described. For instance, *Prevotella* was reduced and *Streptococcus* increased in schizophrenia, while *Schlegelella* appeared uniquely in mania. No consistent oral microbiota alterations were observed in MDD (142). Comparative analyses between MDD and schizophrenia further noticed overlapping shifts in taxa related to inflammation and SCFA/GABA metabolism, alongside disorder-specific alterations (143–146).

Taken together, this review indicates that recurring microbial signatures are reported across multiple psychiatric disorders, while substantial variability persists across studies. Methodological heterogeneity, geographic and dietary factors, and clinical confounders all contribute to this variability. Explicitly acknowledging these sources of heterogeneity is essential for refining study design, improving comparability, and facilitating the translation of microbiota research toward more precise and individualized psychiatric approaches.

### 5.1 Psychotropic medications and microbiota modulation

Emerging evidence suggests that psychotropic medications themselves can significantly modulate gut microbiota composition. This is an important consideration, as medication effects may act as hidden confounders in microbiota-psychiatry research. It may also represent new opportunities for microbiota-targeted therapies.

#### 5.1.1 Selective serotonin reuptake inhibitors

Some SSRIs exhibit direct antimicrobial activity *in vitro* and may induce perturbations of the gut microbiota in ex vivo/SHIME^®^ models. In a SHIME^®^ gut model, SSRI-associated alterations (changes in community structure, reduction of certain beneficial taxa) were partially reversed by a multi-strain probiotic intervention. Thus, it improved the SCFA production, epithelial barrier function, and the inflammatory markers (147).

#### 5.1.2 Vortioxetine

Unlike classical SSRIs, vortioxetine is a multimodal antidepressant with unique receptor activity, including 5-HT_7_ receptor antagonism and modulation of other serotonin receptors (148). A clinical study by showed that vortioxetine treatment was associated with an increased Firmicutes/Bacteroidetes ratio, a marker often linked to improved metabolic and immune balance (149). Importantly, beneficial taxa such as Lachnospira, Roseburia, and Faecalibacterium were negatively correlated with depressive symptom severity. This suggests that vortioxetine’s clinical benefits may partially involve restoration of SCFA-producing bacteria. This aligns with its known cognitive-enhancing and pro-cognitive properties, raising the possibility that microbiota shifts mediate some of vortioxetine’s neuropsychological effects.

#### 5.1.3 Antipsychotics with antibiotic-like effects

Emerging evidence suggests that certain second-generation antipsychotics (SGAs) can influence gut microbiota composition. In germ-free models, olanzapine does not produce the expected weight gain, and antibiotic depletion attenuates its metabolic effects. This suggests a microbiota-dependent mechanism (150). Similarly, Risperidone alters gut microbiota composition in rodents. Transferring this microbiota to germ-free mice induces weight gain, confirming a causal link between dysbiosis and metabolic effects (151). These findings indicate that part of the metabolic burden of SGAs may be mediated by their impact on gut microbial communities. This complicates interpretation of microbiota shifts in psychiatric case-control studies, where medication exposure is often a major confounder. Moreover, a recent preclinical study (152) directly compared the microbiota effects of lurasidone and olanzapine. Thus, olanzapine disrupted microbial composition and promoted dysbiosis consistent with its metabolic side effects. However, lurasidone induced more favorable microbiota changes, including enrichment of anti-inflammatory taxa. These differences may be related to their distinct receptor-binding profiles, with lurasidone (like vortioxetine) engaging serotonin receptor subtypes that are increasingly implicated in gut-brain signaling. Such receptor-driven microbiota modulation opens new perspectives on how drug pharmacodynamics extend beyond the CNS to the gut ecosystem.

## 6 Limitations

Several limitations of this review should be acknowledged. First, the included studies represent a highly heterogeneous body of literature, with substantial variability in cohort characteristics, sampling strategies, sequencing approaches, and analytical pipelines, which limits direct comparability across studies. Second, many studies relied on relatively small sample sizes, reducing statistical power and increasing susceptibility to bias. Differences in microbiota profiling methodologies, particularly the use of 16S rRNA sequencing versus shotgun metagenomics and variable taxonomic resolution, further complicate the interpretation of reported taxonomic alterations and may partly explain inconsistent findings.

Clinical and lifestyle factors (including diet, geographic background, medication exposure, disease stage, and comorbidities) were variably reported and controlled across studies and act as important confounders, limiting the ability to attribute observed microbiota changes specifically to psychiatric disorders. In addition, the absence of a universally accepted definition of a “healthy” gut microbiota poses a conceptual challenge for cross-study comparisons. Importantly, as the available evidence is largely derived from observational primary studies summarized in reviews, causal inferences cannot be drawn from the reported associations.

Together, these limitations are particularly relevant for taxon-centered syntheses, where methodological and clinical heterogeneity can substantially influence apparent convergence or divergence of microbial signatures across disorders. Addressing these challenges will require larger, well-characterized, and methodologically standardized studies to better clarify the role of the gut microbiota in mental health.

## 7 Conclusion

This review summarizes a growing body of evidence linking gut microbiota composition to psychiatric disorders. Across diagnoses, several microbial alterations were consistently reported, suggesting that some biological processes may be shared across conditions, while substantial variability remains between studies. Recurrent findings included reduced abundance of taxa commonly described as anti-inflammatory or butyrate-producing, such as *Coprococcus*, alongside increased levels of genera often associated with pro-inflammatory profiles (153). These recurring patterns were observed across multiple psychiatric disorders, although their direction and magnitude varied depending on study design and population. At the same time, taxa such as *Lactobacillus* and *Bacteroides fragilis* showed context-dependent associations (154), highlighting the importance of host-related factors including genetics, age, immune status, diet, medication exposure, and clinical context (155). Although the available evidence is largely associative and does not support causal conclusions, the taxon-centered, transdiagnostic approach used here helps organize complex and heterogeneous findings across the literature. By shifting the focus away from individual diagnoses and toward shared microbial patterns, this approach may assist in identifying convergent biological signals that cut across traditional diagnostic boundaries.

Integrating microbial patterns with clinical features and functional domains, such as cognition, affect, and arousal, could further improve understanding of psychiatric heterogeneity. Overall, this review supports moving beyond strictly diagnosis-centered models toward frameworks that consider shared microbial patterns across disorders, while explicitly acknowledging the complexity and variability of microbiota-host interactions.
