# Supplementary material for: A taxon-centered review of bacterial shifts in psychiatric disorders
Source: Front Psychiatry. 2026 Mar 2;17:1702172. doi: 10.3389/fpsyt.2026.1702172 (PMC12989607; doi:10.3389/fpsyt.2026.1702172)
Supplement: Supplementary file 1 [file DataSheet1.pdf]

SUPPLEMENTARY MATERIAL

<u>Supplementary Table 1A (Humans)</u>

Legend: *↑(increased), ↓(decreased), ADHD (Attention deficit hyperactivity disorder), Alzheimer Disease (AD), AN (Anorexia Nervosa), ASD (Autism Spectrum Disorder), BD (Bipolar Disorder), OCD (Obsessive-Compulssive Disorder), PTSD (Post Traumatic Stress Disorder), SCZ (Schizophrenia), GWAS (Genome-Wide Association Study), MWAS (Microbiome-Wide Association Study)*

Bacterium	Condition	Direction of change	Study/Cohort Type	Treatment status	Reference
Actinobacteria	Depression	↑	Review Article	Not Applicable	(19).
	AD	↑	Review Article	Not Applicable	(20).
	ASD	↓	Review Article	Not Applicable	(19).
Actinomycetota	PTSD	↓	Review Article	Not Applicable	(11).
	ADHD	↑	Review Article	Not Applicable	(11).
	Depression	↑	Review Article	Not Applicable	(11).
Agathobacter genera	OCD	↓	Review Article	Not Applicable	(20).
Akkermansia genus	Depression	↓	Review Article	Not Applicable	(11).
Alistipes spp.	Depression	↑	Review Article	Not Applicable	(149).
	AD	↓	Review Article	Not Applicable	(20).
Anaerococcus genera	SCZ	↑	Review Article	Not Applicable	(20) (37)
Anaerofilum genera	ASD	↑	Review Article	Not Applicable	(20).
Anaeromassillibacillus genera	Social Anxiety	↑	Review Article	Not Applicable	(11).
Anaerostipes genera	Depression	↑	Review Article	Not Applicable	(49).
	OCD	↓	Review Article	Not Applicable	(20).
Ascomycota (fungi)	SCZ	↑	Review Article	Not Applicable	(127).

Atopobium genera	Depression	↑	Review Article	Not Applicable	(20).
	BD	↑	Review Article	Not Applicable	(20).
Bacillota phyla	Depression	↓	Review Article	Not Applicable	(11).
Bacteroidaceae family	AN	↑ (the risk)	Mendelian randomization (GWAS-based)	Not Applicable	(63).
	BD	Protective Role	Mendelian randomization (GWAS-based)	Not Applicable	(63).
	SCZ	↑	Review Article	Not Applicable	(29).
Bacteroidales spp.	Depression	↑	Review Article	Not Applicable	(29).
Bacteroides fragilis	AD	↓	Review Article	Not Applicable	(20).
Bacteroides Spp.	AD	↓	Review Article	Not Applicable	(20).
	AN	↑ (the risk)	Mendelian randomization (GWAS-based)	Not Applicable	(63).
	Anxiety	↑	Review Article	Not Applicable	(29).
	ADHD	↓	Review Article	Not Applicable	(20).
	BD	↑	Review Article	Not Applicable	(20).
	ASD	↑	Review Article	Not Applicable	(19).
Bacteroides vulgatus	ASD	↑	Review Article	Not Applicable	(148).
Bacteroidetes Phylum	Depression	↑	Review Article<br><br>Case - Control	Not Applicable<br><br>Mixed	(33)<br><br>(132)
	AD	↑(127), ↓ (20)	Review Article	Not Applicable	(20) (127)
	ASD	↓	Review Article	Not Applicable	(19).
Bacteroidota phyla	Depression	↑	Review Article	Not Applicable	(11).

Barnesiella intestinihominis	ASD	↑	Review Article	Not Applicable	(20).
Betaproteobacterium	BD	↑	Review Article	Not Applicable	(20).
Bifidobacterium animalis lactis*	Depression	as probiotics → reduces stress, anxiety and Depression	Review Article	Not Applicable	(10).
Bifidobacterium bifidum	SCZ	as probiotics → improves PANSS	Review Article	Not Applicable	(10).
	Depression	as probiotics → Improved Depression scores with L. acidophilus and L. casei	Review Article	Not Applicable	(127).
	AD	as probiotics →improving MMSE scores in AD	Review Article	Not Applicable	(20).
Bifidobacterium longum	Depression	↓<br>as probiotics → Improved Depression scores	Intervention study<br>Review Article	Drugs Free<br>Not Applicable	(57).<br>(127).

Bifidobacterium Spp.	ASD	↓	Review Article	Not Applicable	(127) (37)
	Depression	as probiotics → ↓Depression	Review Article	Not Applicable	(19) (136)
	AD	↓	Review Article	Not Applicable	(127).
Blautia spp.	SCZ	↓	Review Article	Not Applicable	(19).
	Depression	↑	Review Article	Not Applicable	(49).
Blautia wexlerae	Food Addiction	↓ at people with high YFAS score	Observational Cohort (cross-sectional)	Not reported	(157).
Burkholderia genera	ASD	↑	Review Article	Not Applicable	(20).
Butyricimonas Spp.	ADHD	Protective Role	"Mendelian randomization (GWAS-based)"	Not Applicable	(63).
Christensenellaceae R7 group	Anxiety	↓	Review Article	Not Applicable	(11).
	AN	↑	Review Article	Not Applicable	(20).
Clostridiaceae (C_1_family)	BD	↓ (the risk)	Mendelian randomization (GWAS-based)	Not Applicable	(63).
Clostridiales order	ASD	↑	Review Article	Not Applicable	(20).
	Anxiety and Depression	↓	Review Article	With antidepressants and anxiolitics	(137).
	ADHD	↓	Review Article	Not Applicable	(20).

Clostridium butyricum	Depression	as probiotics → Adjunct therapy improved treatment-resistant Depression	Review Article	Not Applicable	(127).
	OCD	↑	Review Article	Not Applicable	(20).
Clostridium cluster IV	BD	↑	Review Article	Not Applicable	(20).
Clostridium perfringens	ASD	↑	Review Article	Not Applicable	(20) (37)
Clostridium spp.	ASD	↑	Review Article	Not Applicable	(127) (37)
	SCZ	↓	Case-control	Medication washout (7 days before sampling)	(145).
Collinsella aerofaciens	ADHD	↓	Review Article	Not Applicable	(20).
Collinsella spp.	BD	↑	Review Article	Not Applicable	(19) (20)
	SCZ	↑	Review Article	Not Applicable	(19) (20)
	ADHD	↑	Review Article	Not Applicable	(19) (20)

Coprococcus spp.	Depression	↓	Review Article<br><br>Systematic Review and meta-analysis (human studies)<br><br>Population-based human cohort (MWAS)	Not Applicable<br><br>Mixed (only 2 studies are drug-naive)<br><br>Antidepressant medication excluded	(20)<br><br>(135)<br><br>(138)
	OCD	↓	Review Article	Not Applicable	(20).
	SCZ	↓	Review Article	Not Applicable	(20).
	BD	↓	Review Article	Not Applicable	(20).
Coriobacteriaceae family	SCZ	↑	Review Article	Not Applicable	(20).
	AD	↓	Review Article	Not Applicable	(20).
	BD	↑	Review Article	Not Applicable	(20) (38)
Cyanobacteria phylum	Anxiety	Asociate	Mendelian randomization (GWAS-based)	Not Applicable	(63).
Deferribacteres family	OCD	↑	Review Article	Not Applicable	(20).
Desulfovibrio spp.	ASD	↑	Review Article	Not Applicable	(19) (37)
Dialister spp.	Depression	↓	Review Article	Not Applicable	(15) (20)
Dorea genera	AD	↑			(34).
	ASD	↑	Review Article	Not Applicable	(20).

Eggerthella lenta	Depression	↑	Review Article<br>Population-based human cohort (MWAS)	Not Applicable<br><br>Antidepressant medication excluded	(19)<br>(138)
Enterobacteriaceae	Depression	↑	Review Article	Not Applicable (19)<br>but Mixed in (106)	(19).<br><br>(106).
	ASD	↑	Review Article	Mixed	(106).
Enterococcus faecium	ADHD	↓	Review Article	Not Applicable	(20).
Erysipelotrichaceae family	OCD	↑	Review Article	Not Applicable	(20).
Erysipelotrichaceae UCG003 genus	BD	↓ (the risk)	Mendelian randomization (GWAS-based)<br><br>Case-Control	Not Applicable<br><br><br>Medication washout<br>(7 days before sampling)	(63)<br><br><br>(145)
Escherichia / Shigella	Depression	↓	Review Article	Not Applicable	(20).
Escherichia coli	ASD	↓	Review Article	Not Applicable	(20).
	SCZ	↓	Review Article	Not Applicable	(19).
Eubacterium rectale	AD	↓	Review Article	Not Applicable	(17) (20)

Faecalibacterium spp.	Depression	↓	Review Article<br>Review Article<br>Systematic Review and meta-analysis (human studies)	Not Applicable<br>Not Applicable<br>Mixed (only 2 studies are drug-naive)	(20)<br>(126)<br>(135)
	BD	↑	Review Article	Not Applicable	(20).
	ASD	↓	Review Article	Not Applicable	(20).
Firmicutes	Depression	↓	Review Article	Not Applicable	(19) (29) (33) (127) (136)
	BD	↓	Review Article	Not Applicable	(37).
	AD	↓	Review Article	Not Applicable	(149).
Firmicutes/Bacteroidetes ratio	ASD	↑	Review Article	Not Applicable	(19).
Flavonifractor genera	BD	↑	Review Article	Not Applicable	(49).
	Depression	↑	Review Article	Not Applicable	(20).
Fusicatenibacter	Anxiety	↓	Review Article	Not Applicable	(11).
Fusobacteria phyla	Depression	↑	Review Article	Not Applicable	(11).
Gelria genera	Depression	↑	Review Article	Not Applicable	(6).
Gordonibacter genera	Social Anxiety	↑	Review Article	Not Applicable	(11).
Haemophilus genera	SCZ	↓	Review Article	Not Applicable	(20) (37)
Holdemania genera	Depression	↑	Review Article	Not Applicable	(6).
Klebsiella genera	Depression	↑	Review Article	Not Applicable	(20).
	SCZ	↑	Review Article	Not Applicable	(20).
Lachnospira genus	Depression	↓	Review Article	Not Applicable	(11).

Lachnospiraceae family	Depression	↓	Review Article	Not Applicable	(33) (29)
	AD	Altered	Review Article	Not Applicable	(127).
	ADHD	↓	Review Article	Not Applicable	(20).
	OCD	↑	Review Article	Not Applicable	(20).
	SCZ	↓	Review Article<br><br>Case-control	Not Applicable<br><br>Not controlled	(20)<br><br>(143)
Lachnospiraceae incertae sedis	Depression	↑	Review Article	Not Applicable	(20).
Lachnospiraceae ND3007	ASD	↓ (the risk)	Mendelian randomization (GWAS-based)<br><br>Case-Control	Not Applicable<br><br>Medication washout (7 days before sampling)	(63)<br><br>(145)
Lactobacillus acidophilus	Depression	as probiotics → Improved Depression scores with B. bifidum and L. casei	Review Article	Not Applicable	(127).
	Anxiety	Part of multi-strain probiotic with anxiolytic effects*	Review Article	Not Applicable	(127).

Lactobacillus bulgaricus	Anxiety and Depression	as probiotics → Improved anxiety and depression	Review Article	Not Applicable	(10) (12)
Lactobacillus casei	Depression	as probiotics → Improved Depression scores with L. acidophilus and B. bifidum	Review Article	Not Applicable	(127).
Lactobacillus fermentum	SCZ	as probiotics → improves SCZ	Review Article	Not Applicable	(19).
Lactobacillus group.	ASD	↓	Review Article	Not Applicable	(20).
	AD	↓	Review Article	Not Applicable	(17).
	BD	↑	Review Article	Not Applicable	(20).
	Depression	↓	Case–control (first episode psychosis)<br>Review Article	Mixed (+/- antipsychotics)<br>Not Applicable	(130)<br>(8) (149)
Lactobacillus helveticus	Anxiety and Depression	↓	Review Article	Not Applicable	(10).
	Depression	as probiotics → Improved Depression scores	Review Article	Not Applicable	(127).

Lactobacillus plantarum	Anxiety	as probiotics → Improved anxiety	Review Article	Not Applicable	(19).
Lactobacillus reuteri	SCZ	as probiotics + vitamin D → improves PANSS	Review Article	Not Applicable	(10).
Lentisphaerae phylum	PTSD	↓	Review Article	Not Applicable	(11).
Marvinbryantia genus	SCZ	↓ (the risk)	Mendelian randomization (GWAS-based)	Not Applicable	(63).
Megamonas genus	Depression	↓	Review Article	Not Applicable	(11).
Megasphaera genera	SCZ	↑	Review Article	Not Applicable	(20).
Methanobrevibacter genera	SCZ	↑	Review Article<br>Case-sectional	Not Applicable<br>Medicated (stable antipsychotic treatment ≥6 months)	(20)<br>(142)
Methanobrevibacter smithii	AN	↑	Review Article	Not Applicable	(20).
Odoribacter genera	OCD	↓	Review Article	Not Applicable	(20).
Oscillibacter spp.	Depression	↑	Review Article<br>Case-sectional	Not Applicable<br>Mixed	(20)<br>(146)
Oscillospira spp.	ASD	↓	Review Article	Not Applicable	(20).
	OCD	↓	Review Article	Not Applicable	(20).
Parabacteroides genera	AD	↓	Review Article	Not Applicable	(20).
	Depression	↑	Review Article	Not Applicable	(20).
	ADHD	↓	Review Article	Not Applicable	(20).

Paraprevotella genera	AD	↓	Review Article	Not Applicable	(20).
	Depression	↑	Review Article	Not Applicable	(6).
Parasutterella excrementihominis	ASD	↓	Review Article	Not Applicable	(20).
Parasutterella genera	Depression	↑	Review Article	Not Applicable	(20).
Phascolarctobacterium	Depression	↑	Review Article	Not Applicable	(49).
Prevotella spp.	Depression	↓	Review Article	Not Applicable	(6) (19) (135)
	SCZ	↑	Review Article	Not Applicable	(19).
Prevotellaceae family	SCZ	↑	Review Article	Not Applicable	(38).
	OCD	↓	Review Article	Not Applicable	(20).
	Anxiety and Depression	↓	Review Article	Not Applicable	(19).
Prevotellaceae NK3B31	Depression	↓	Review Article	Not Applicable	(11).
Proteobacteria	Depression	↑	Review Article	Not Applicable	(19).
	OCD	↑	Review Article	Not Applicable	(20).
	SCZ	↓	Review Article	Not Applicable	(20).
Pseudomonadota phylum	Depression	↑	Review Article	Not Applicable	(11).
Psychrobacter genera	Depression	↓	Review Article	Not Applicable	(33).
Rikenellaceae family	OCD	↑	Review Article	Not Applicable	(20).
Rombutsia genera	ADHD	↓	Review Article	Not Applicable	(20).

Roseburia genus	Depression	Altered	Review Article	Not Applicable	(49).
	ADHD	↑	Review Article<br>Mendelian randomization (GWAS-based)	Not Applicable	(20)<br>(63)
	BD	↓	Review Article	Not Applicable	(20).
	ASD	↓	Review Article	Not Applicable	(20).
	SCZ	↓	Review Article	Not Applicable	(20).
	AN	↓	Review Article	Not Applicable	(20) (149)
Ruminiclostridium 5 genus	SCZ	↑ (the risk)	Mendelian randomization (GWAS-based)	Not Applicable	(63).
Ruminococcaceae family	Depression	↓	Review Article<br>Cross-Sectional<br>Population-based human cohort (MWAS)	Not Applicable<br>Not controlled<br><br>Antidepressant medication excluded	(33)<br>(137)<br>(138)
	AD	↓	Review Article	Not Applicable	(20).
	OCD	↑	Review Article	Not Applicable	(20).
	SCZ	Altered	Case-Control	Not controlled	(143).
Ruminococcaceae UCG005 genus	ASD	↑ (the risk)	Mendelian randomization (GWAS-based)	Not Applicable	(63).

Ruminococcaceae UCG010 genus	Anxiety	↑ (the risk)	Mendelian randomization (GWAS-based)	Not Applicable	(63).
Ruminococcus gnavus	Anxiety	↑	Review Article	Not Applicable	(19).
Ruminococcus spp.	Depression	↓	Review Article	Not Applicable	(20) (33)
	BD	↓	Review Article	Not Applicable	(20).
	SCZ	↓	Review Article	Not Applicable	(20).
	AN	↓	Review Article	Not Applicable	(149).
	ADHD	↑	Review Article	Not Applicable	(20).
Selenomonadales Spp.	AD	Altered	Review Article	Not Applicable	(127).
Streptococcus thermophilus	Depression	as probiotics → improves mood	Review Article	Not Applicable	(10).
Subdoligranulum genus	Depression	↓	Review Article<br>Population-based human cohort (MWAS)	Not Applicable<br><br>Antidepressant medication excluded	(11)<br>(138)
	ASD	↓	Review Article	Not Applicable	(19) (20)
	Anxiety	↑	Review Article	Not Applicable	(19).
Succinivibrio genera	SCZ	↑	Review Article	Not Applicable	(20).

Sutterella	ASD	↑	Review Article	Not Applicable	(43).
	Anxiety compared to depression not to healthy individuals.	↑	Review Article	Not Applicable	(11)
	AD	↓	Review Article	Not Applicable	(20).
	SCZ	↑	Case-Control	Medication washout (7 days before sampling)	(145).
Tenericutes phylum	OCD	↑	Review Article	Not Applicable	(20).
Turicibacter genera	Depression	↑	Review Article	Not Applicable	(6).
	ASD	↓	Review Article	Not Applicable	(20).
Turicibacter sanguinis	ASD	↑	Review Article	Not Applicable	(20).
Veillonellaceae family	SCZ	↑	Review Article	Not Applicable	(20) (38)
Verrucomicrobia Spp.	AD	Altered	Review Article	Not Applicable	(20).
	ASD	Altered	Review Article	Not Applicable	(10).
Verrucomicrobiota	PTSD	↓	Review Article	Not Applicable	(11).

Supplementary Table 1B (Animals)

Legend: ↑*(increased),* ↓*(decreased), Alzheimer Disease (AD), ASD (Autism Spectrum Disorder),*

**Bacterium**	**Condition**	**Direction of change**	**Species**	**Model**	**Study Type**	**Reference**
Bacteroides fragilis	ASD-like	As probiotics → Improved of ASD-like behaviors	Mouse	MIA (maternal immune activation)	Review Article	(127).
Bifidobacterium breve	Anxiety-like and Depression-like	As probiotics → reduced anxiety- and depressive-like behaviors	Mouse/Rat	stress-related /anxiety models	Review Article	(127).
Bifidobacterium Spp.	Anxiety-like and Depression-like	As probiotics → reduced anxiety- and depressive-like behaviors	Mouse/Rat	stress-related /anxiety models	Review Article	(127).
Blautia Genus	Food addiction-like behavior	↓	Mouse	C57BL/6J Food addiction–like behavior model	Experimental Mouse Study	(157).

Campylobacter jejuni	Anxiety-like	↑ anxiety-like behavior<br><br>Increased neuronal activation (c-Fos expression) in brain regions involved in autonomic control and stress responses	Mouse	anxiety models + acute gastrointestinal infection	Review Article	(17)<br>(43)
Citrobacter rodentium	Anxiety-like	↑ anxiety-like behavior<br>Effects likely mediated by vagal sensory neurons	Mouse	murine colonic hyperplasia	Review Article	(17)<br>(43)
Clostridiales order	Depression-like	↓	Rat	Chronic Variable Stress (CVS)-induced depression	Review Article	(33)<br>(137)
Corynebacterium genera	Depression-like	↓	Rat	Chronic Variable Stress (CVS)-induced depression	Review Article	(33).

Enterobacteriaceae	Anxiety-like and Depression-like	↑ associated with reduced levels of Lactobacillus, reduced hippocampal GABA_A receptor subunit (α5, δ) expression and altered limbic regulation.	Mouse/Rat	Early-life gut X microbiota disturbance	Review Article	(107).
Lactobacillus acidophilus	AD-like	As part of multistrain probiotics → Improvement of memory and learning deficits in AD models	Mouse/Rat	stress-related / murine models	Review Article	(127).
Lactobacillus plantarum*	AD	As probiotics → reduced anxiety- and depressive-like behaviors + cognitive improvements	Mouse/Rat	stress or neurodevelopmental models	Review Article	(127).
Lactobacillus rhamnosus	Anxiety-like	As probiotics → Improved anxiety	Mouse	Balb/c male stress-related /anxiety models	Review Article	(127).

Peptostreptococcaceae incertae	Depression-like	↓	Rat	Chronic Variable Stress (CVS)-induced depression	Review Article	(33).
Psychrobacter genera	Depression-like	↓	Rat	Chronic Variable Stress (CVS)-induced depression	Review Article	(33).
Veillonella spp.	ASD-like	↓	Mouse	Shank3 transgenic mice (autism candidate gene)	Review Article	(127).
Prevotella spp.	ASD-like	↓	Mouse	Shank3 transgenic mice (autism candidate gene)	Review Article	(127).
Lactobacillus spp	ASD-like	↓	Mouse	Shank3 transgenic mice (autism candidate gene)	Review Article	(127).
